# Multi-layered epigenetic regulation of *IRS2* expression in the liver of obese individuals with type 2 diabetes

**DOI:** 10.1007/s00125-020-05212-6

**Published:** 2020-07-24

**Authors:** Christin Krause, Cathleen Geißler, Heidi Tackenberg, Alexander T. El Gammal, Stefan Wolter, Joachim Spranger, Oliver Mann, Hendrik Lehnert, Henriette Kirchner

**Affiliations:** 1 First Department of Medicine, Division of Epigenetics and Metabolism, University of Lübeck, Ratzeburger Allee 160, 23562 Lübeck, Germany; 2 Department of General, Visceral and Thoracic Surgery, University Medical Centre Hamburg-Eppendorf, Hamburg, Germany; 3 Department of Endocrinology and Metabolism, Charité - Universitätsmedizin Berlin, Berlin, Germany; 4 German Center for Diabetes Research (DZD), München-Neuherberg, Germany

**Keywords:** DNA methylation, Human liver, IRS2, MicroRNA let7e, NAFLD, Type 2 diabetes

## Abstract

**Aims/hypothesis:**

IRS2 is an important molecular switch that mediates insulin signalling in the liver. *IRS2* dysregulation is responsible for the phenomenon of selective insulin resistance that is observed in type 2 diabetes. We hypothesise that epigenetic mechanisms are involved in the regulation of *IRS2* in the liver of obese and type 2 diabetic individuals.

**Methods:**

DNA methylation of seven CpG sites was studied by bisulphite pyrosequencing and mRNA and microRNA (miRNA) expression was assessed by quantitative real-time PCR in liver biopsies of 50 obese non-diabetic and 31 obese type 2 diabetic participants, in a cross-sectional setting. Methylation-sensitive luciferase assays and electrophoretic mobility shift assays were performed. Furthermore, HepG2 cells were treated with insulin and high glucose concentrations to induce miRNA expression and *IRS2* downregulation.

**Results:**

We found a significant downregulation of *IRS2* expression in the liver of obese individuals with type 2 diabetes (0.84 ± 0.08-fold change; *p* = 0.0833; adjusted *p* value [*p*_a_] = 0.0417; *n* = 31) in comparison with non-diabetic obese participants (*n* = 50). This downregulation correlated with hepatic *IRS2* DNA methylation at CpG5. Additionally, CpG6, which is located in intron 1 of *IRS2*, was hypomethylated in type 2 diabetes; this site spans the sterol regulatory element binding transcription factor 1 (SREBF1) recognition motif, which likely acts as transcriptional repressor. The adjacent polymorphism rs4547213 (G>A) was significantly associated with DNA methylation at a specificity-protein-1 (SP1) binding site (CpG3). Moreover, DNA methylation of cg25924746, a CpG site located in the shore region of the *IRS2* promoter-associated CpG island, was increased in the liver of individuals with type 2 diabetes, as compared with those without diabetes. A second epigenetic mechanism, upregulation of hepatic miRNA hsa-let-7e-5p (let-7e-5p) in obese individuals with type 2 diabetes (*n* = 29) vs non-diabetic obese individuals (*n* = 49) (1.2 ± 0.08-fold change; *p* = 0.0332; *p*_a_ = 0.0450), is likely to act synergistically with altered *IRS2* DNA methylation to decrease *IRS2* expression. Mechanistic in vitro experiments demonstrated an acute upregulation of let-7e-5p expression and simultaneous *IRS2* downregulation in a liver (HepG2) cell line upon hyperinsulinaemic and hyperglycaemic conditions.

**Conclusions/interpretation:**

Our study highlights a new multi-layered epigenetic network that could be involved in subtle dysregulation of *IRS2* in the liver of individuals with type 2 diabetes. This might lead to fine-tuning of *IRS2* expression and is likely to be supplementary to the already known factors regulating *IRS2* expression. Thereby, our findings could support the discovery of new diagnostic and therapeutic strategies for type 2 diabetes.

**Figure:**
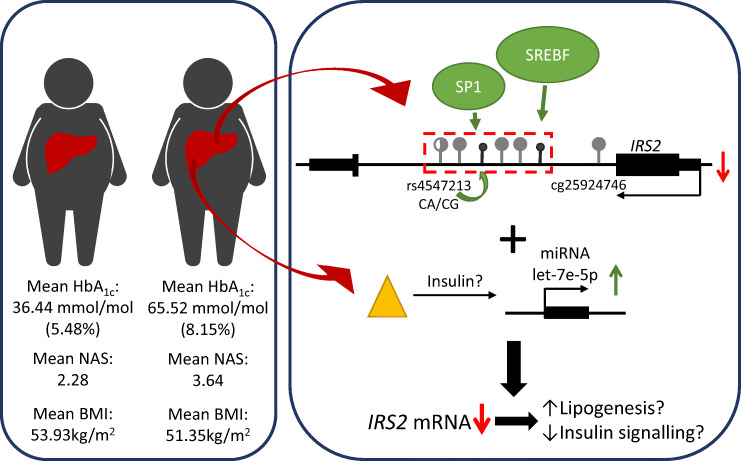
Graphical abstract

**Electronic supplementary material:**

The online version of this article (10.1007/s00125-020-05212-6) contains peer-reviewed but unedited supplementary material, which is available to authorised users.

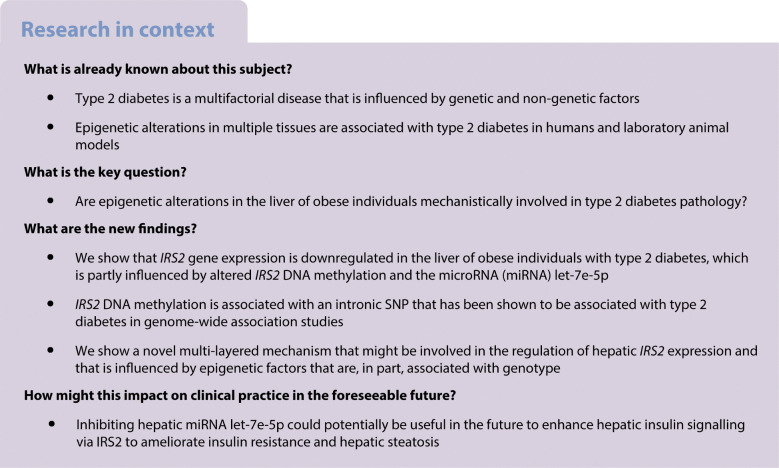



## Introduction

IRS2 is one of the most important molecular switches mediating hepatic insulin receptor signalling and is involved in the phenomenon of selective insulin resistance, in which the lipogenic actions of insulin remain intact while glucose uptake and glycogen synthesis are disrupted [1, 2]. Consequently, mice lacking *Irs2* exhibit selective insulin resistance [2] and specific knockout of *Irs2* in hepatocytes activates gluconeogenic pathways [3]. Moreover, leptin-deficient *ob/ob* mice show a downregulation of *Irs2* expression simultaneously with increased expression of the gene encoding sterol regulatory element binding transcription factor 1 (*Srebf1*) [4]. Thus, we hypothesise that *IRS2* dysregulation plays a key role in the manifestation of type 2 diabetes, as well as liver steatosis, in humans. Previously it was shown that *IRS2* expression is altered in obese individuals and in people suffering from non-alcoholic fatty liver disease (NAFLD) [5, 6]; however, it currently remains unclear as to whether *IRS2* contributes to the pathogenesis of type 2 diabetes and fatty liver disease in humans, and the mechanisms responsible for *IRS2* dysregulation are unknown.

Epigenetic marks are regulated by environmental factors and their contribution to regulatory mechanisms of metabolic diseases is only starting to be unravelled. Aberrant expression of microRNAs (miRNAs) and changes in DNA methylation at CpG dinucleotides are responsible for the highly dynamic influence of environmental factors and lifestyle on gene expression, which can be passed on throughout generations [7]. The miRNA family hsa-let-7 is likely to regulate *IRS2* expression and is associated with changes in glucose homeostasis in the muscle and brain [8, 9]. DNA methylation at cytosines of CpG oligonucleotides influences gene expression, for example, by modulating the binding of transcription factors to the DNA [10]. Previously, a 3% increase in *IRS2* DNA methylation within a CpG island of exon 1 (cg05514401) was identified in subcutaneous adipose tissue of 15 insulin-resistant obese women concomitant with decreased *IRS2* gene expression, as compared with 14 non-obese normoglycaemic women [11], indicating that *IRS2* expression may be epigenetically regulated in metabolic diseases. Moreover, in a large-scale epigenome-wide association study (EWAS), in blood samples, DNA methylation of the CpG site cg25924746, which is located in the shore region of the *IRS2*-related CpG island, was associated with type 2 diabetes [12]. However, functional consequences of these alterations in *IRS2* DNA methylation remain unclear and whether they could lead to future glucose-lowering therapies is not yet known.

Our study focuses uniquely on mechanisms leading to altered *IRS2* expression in the liver of individuals with diabetes, as compared with non-diabetic participants, independent of classical risk factors, such as age, sex and obesity.

## Methods

### Study design and participants

Liver wedge biopsies from segment III of the liver were obtained from 81 obese (BMI >32 kg/m^2^) male and female participants during bariatric surgery at the University Hospital Eppendorf (UKE; Hamburg, Germany), as previously described [13], in a cross-sectional study design. Blood samples for serum extraction were also drawn on the day of surgery, after an overnight fast. The study was approved by the local ethics committee of the Ärztekammer Hamburg (PV4889) and all participants gave informed consent (see electronic supplementary material [ESM] Methods).

Participants were categorised by clinical examination and use of glucose-lowering medication into type 2 diabetes (*n* = 31) and non-diabetic control (*n* = 50) groups (ESM Table 1). All participants in the type 2 diabetes group were taking glucose-lowering medication. NAFLD activity score (NAS) was determined during surgery according to the current recommendations by two expert pathologists. For selected sub-analysis, this score was used for the stratification of individuals with (NAS = 1–6) and without (NAS = 0) liver steatosis and fibrosis. Clinical parameters were measured at the day of the surgery at the Institut für Klinische Chemie und Laboratoriumsmedizin, Zentrum für Diagnostik, Universitätsklinikum Eppendorf, Hamburg, Germany, according to the DIN EN ISO 15189:2014 certification. Glucose, cholesterol, HDL, aspartate aminotransferase (AST), alanine aminotransferase (ALT) and triacylglycerols were determined using photometric assays (kinetic bichromatic analysis; in-house assay by Institut für Klinische Chemie und Laboratoriumsmedizin, UKE, Germany). HbA_1c_ was quantified by capillary electrophoresis or by turbidimetric inhibition assays (in-house assay by Institut für Klinische Chemie und Laboratoriumsmedizin, UKE).

All samples were analysed in randomised order. Randomisation was achieved by processing the samples in the order of the biobank identification number, which was not in order or associated with the two study groups. Sample preparation and processing was blinded as all samples were only labelled with the biobank identification number. Data analysis was not blinded. Transcription-factor binding profiles were obtained from the JASPAR database [14].

### DNA methylation measurement

DNA was extracted from 25 mg of snap frozen liver using the QIAmp mini kit (QIAGEN, Hilden, Germany). Genomic DNA was bisulphite-converted (bisDNA) using the EpiTectFast Bisulfite kit (QIAGEN). Bisulphite PCR was performed with the PyroMark PCR kit (QIAGEN). One primer pair was used to analyse CpG sites 1–5, a second primer pair was used to analyse CpG site 6 and a third pair was used for analysis of cg25924746 (see ESM Table 2 for a list of primers, including primer for rs4547213). DNA methylation was measured by bisulphite pyrosequencing using PyroMark Q48 and PyroMark Q48 Advanced reagents (QIAGEN) (see ESM Methods).

### RNA isolation and gene expression analysis

Total RNA was extracted from 25 mg of snap frozen liver using the Mirneasy mini kit (QIAGEN) and quantified spectrometrically. RNA (2 μg) was reverse transcribed into cDNA using the SuperScript VILO cDNA synthesis kit (Invitrogen, Carlsbad, CA, USA).

Gene expression was measured in duplicates using TaqMan assays (Applied Biosystems, Foster City, CA, USA) and calculated with the ΔΔC_t_ method. The following genes were analysed: *IRS2*, *PCK1*, *G6PC*, *PDK1*, *ACACA*, *ACACB*, *FASN*, *SCD*, *PNPLA2*, *FOXO1*, *ELOVL6*. Gene expression was normalised to *CASC3* expression (ESM Methods).

For hepatic miRNA cDNA synthesis, 10 ng of total RNA was reverse transcribed with the TaqMan Advanced miRNA cDNA synthesis kit (Applied Biosystems). Expression of hsa-let-7e-5p (herein referred to as let-7e-5p) miRNA was measured in duplicates using TaqMan Advanced miRNA assays and calculated using the ΔΔC_t_ method, with hsa-miR-24-3p being used as the housekeeping gene (ESM Methods).

### Measurement of serum miRNA concentrations

Serum miRNA was extracted using the recommended spike-in control cel-miR-39-3p approach (miRNeasy Serum/Plasma Advanced kit; QIAGEN). MiRNAs were reverse transcribed with the qScript miRNA cDNA synthesis kit (QuantaBio, Beverly, MA, USA). Gene expression of let-7e-5p was measured by qPCR in duplicates using the FastStart Universal SYBR Green Master (Roche, Basel, Switzerland), self-designed qPCR primers (ESM Table 2) and a universal primer from the qScript kit. Expression was normalised to the spike-in control cel-miR-39-3p expression using the ΔΔC_t_ method (see ESM Methods).

### Methylation-sensitive luciferase reporter gene assay

Luciferase plasmids with a CpG-free backbone and CpG-free cytomegalovirus (CMV) promoter (pCpGL-CMV-Fluc [15]) were created with different inserts (CpG1; rs4547213; CpG1–3; CpG1–6; rs4547213 and CpG2–6; ESM Table 2). Consequently, plasmids were either in vitro methylated using SssI methylase (NEB, Ipswich, MA, USA) or mock methylated (incubation without SssI). HepG2 cells were purchased from ATCC (Manassas, VA, USA) and were mycoplasma free. HepG2 were co-transfected with 100 ng luciferase-reporter plasmid and either 500 ng β-galactosidase control plasmid (pCMV-bGal) or 10 ng SV40 *Renilla* control plasmid (pRL-SV40) and luciferase activity was measured after 24 h of incubation (Promega, Madison, WI, USA). The luciferase assays were each performed three times, in triplicate (see ESM Methods).

### Electrophoretic mobility shift assay

Binding reactions containing HepG2 nuclear extracts (NE-PER Nuclear and Cytoplasmatic Extraction Reagents, ThermoFisher, Waltham, MA, USA) and biotinylated oligonucleotides were incubated prior to gel (Invitrogen) loading. For supershift assays, nuclear extracts were incubated with specific antibodies (polyclonal IgG α SREBF1 [PA1-46142] and polyclonal IgG α specificity-protein-1 [SP1; PA5-29165]; both Invitrogen). Protein-DNA complexes were plotted on nylon membranes and detected via chemiluminescence using the Chemiluminescent Nucleic Acid Detection Module (Thermo Fisher). The competitive erasure of protein binding to *IRS2* was repeated five times to test for specificity of protein binding. Incubation with SREBF1 and SP1 antibodies was repeated three times. The electrophoretic mobility shift assay (EMSA) to determine the effect of SNP rs4547213 on protein binding was performed twice. One representative blot is shown (see ESM Methods).

### Insulin treatment of HepG2 cells

HepG2 cells were cultivated in high glucose (25 mmol/l) DMEM supplemented with 0.5% (wt/vol.) BSA and insulin (100 nmol/l or 500 nmol/l Actrapid; NovoNordisk, Bagsværd, Denmark) or in low glucose (8.3 mmol/l) DMEM supplemented with 0.5% (wt/vol.) BSA. After 24 h of treatment, miRNA-cDNA and mRNA-cDNA synthesis steps were performed, as described above. The experiment was repeated three times and conducted in duplicates each time (see ESM Methods).

### General statistics

Data are expressed as means±SEM. Gene expression data was normalised to the respective control group. Results were either visualised as mean fold±SEM or normalised by min/max normalisation to a scale from 0 (minimum expression) to 1 (maximum expression) for correlation analysis. Differences in DNA methylation were tested by two-sided Wilcoxon rank-sum test and differences in gene expression by two-sided Student’s *t* test, or one-way ANOVA if more than two groups were compared, with a post hoc test to compare individual means. (GraphPad Prism, version 7; GraphPad Software, La Jolla, CA, USA). Post hoc testing was performed by comparing each mean value to the mean of the control condition. Post hoc-calculated *p* values were adjusted for multiple testing by the false discovery rate (FDR) method of Benjamini and Hochberg and a *q* < 0.05 was assumed as significant. Spearman’s correlation was used for ordinal values. For continuous variables, Pearson’s correlation was calculated (MATLAB R2018a; The MathWorks, Natick, MA, USA). Significance was assumed at *p* < 0.05 (see ESM Methods).

For association analysis between the polymorphism and the incidence of type 2 diabetes, a logistic regression analysis assuming age, sex and BMI as confounding factors was performed (MATLAB R2018a). Because age and sex was different between the non-diabetic and type 2-diabetic groups, multiple linear regression models using age, sex and BMI as cofactors were used to analyse gene expression and DNA-methylation data (R version 3.5.1; RStudio, Boston, MA, USA). This is indicated by adjusted *p* values (*p*_a_) and all results that are *p*_a_ < 0.05 were considered significant independently of age, sex and BMI (ESM Table 3).

All data points were tested for outliers by the robust regression and outlier (ROUT) method in GraphPad Prism, version 7 and, subsequently, outliers were removed from the analysis. All correlation analyses were corrected for multiple testing using the Benjamini–Hochberg procedure, applying an FDR of 15% (*q* < 0.15) (R version 3.5.1); *q* values are provided in ESM Table 4, and *p* values and *q* values are provided in ESM Table 5.

The effect of a polymorphism on *IRS2* gene expression was assessed by an expression quantitative trait loci (eQTL) analysis, which was performed using a linear mixed-effect model in R, which included age, sex and BMI as cofactors for the calculation of the fitted model [16].

## Results

### Hepatic *IRS2* expression is associated with liver health

**Fig. 1:**
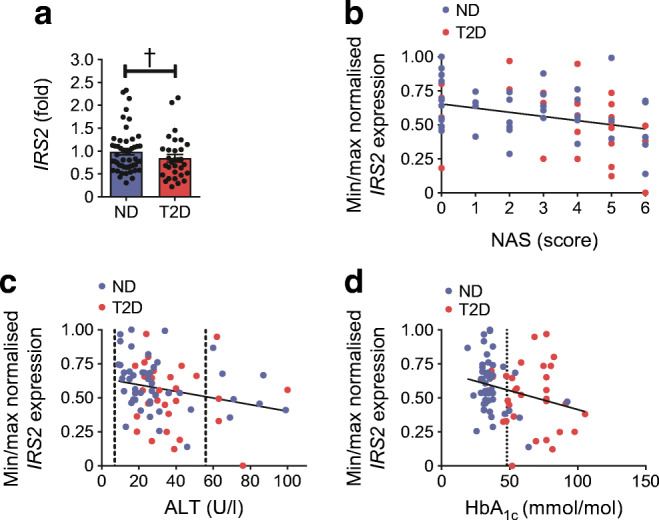
The impact of hepatic *IRS2* expression on liver health status. (**a**) Hepatic *IRS2* expression is significantly decreased in individuals diagnosed with type 2 diabetes (T2D; *n* = 31) in comparison with those without type 2 diabetes (ND; *n* = 50), independently of age, sex or BMI (*p*_a_ = 0.0417). Gene expression was normalised to the expression of a housekeeper gene and is shown as mean fold ± SEM relative to ND, as calculated by the ${2}^{-{\Delta  \Delta  C}_t}$ method. (**b**, **c**) *IRS2* expression correlates with liver steatosis and fibrosis status (NAS) (**b**; *r* = −0.2937, *p*_a_ = 0.0157; *q* = 0.0722; *n* = 68) and with serum concentration of ALT (**c**; *r* = −0.2403, *p*_a_ = 0.0143; *q* = 0.0722; *n* = 78). (**d**) Hepatic *IRS2* expression correlates with HbA_1c_ values independently of age, sex and BMI (*r* = −0.2668, *p*_a_ = 0.0075; *q* = 0.0578; *n* = 68). In (**c**) and (**d**), reference values are indicated by dashed lines. Pearson’s correlation or Spearman’s correlation (*p*) and linear regression analysis (*p*_a_) was used to calculate an association between gene expression (ΔC_t_) and other parameters. For visualisation, gene expression was normalised by minimum (min)/maximum (max) normalisation (1 − (*x*_i_ − *x*_min_)/(*x*_max_ − *x*_min_)), to a scale from 0 (min expression) to 1 (max expression), whereby *x*_i_ is the current ΔCt value of a specific gene, *x*_min_ is the minimum ΔCt value of all measurements of a specific gene and *x*_max_ is the maximum ΔCt value of all measurements of a specific gene. Gene expression is shown as normalised expression on the axes. †*p*_a_ < 0.05

We measured hepatic *IRS2* expression in liver tissue of obese individuals who were stratified by type 2 diabetes, as well as liver fibrosis and steatosis status (NAS). *IRS2* expression was found to be decreased in the liver of obese individuals with type 2 diabetes (*n* = 31) vs obese individuals without type 2 diabetes (*n* = 50) (0.84 ± 0.08-fold change; *p* = 0.0833; *p*_a_ = 0.0417; Fig. 1a). *IRS2* expression correlated significantly with NAS (*p*_a_ = 0.0157; *q* = 0.0722; Fig. 1b) and serum ALT (*p*_a_ = 0.0143; *q* = 0.0722; Fig. 1c). Moreover, *IRS2* expression correlated with HbA_1c_ (*p*_a_ = 0.0075; *q* = 0.0578; Fig. 1d).

To investigate the impact of *IRS2* dysregulation in the diabetic liver, we analysed expression of genes that are downstream of insulin signalling and involved in glucose and lipid metabolism. Hepatic *IRS2* mRNA content directly correlated with gene expression of *ACACA*, *FASN*, *SCD*, *PNPLA2*, *PCK1* and *PDK1* (ESM Fig. 1a-j and ESM Table 5), indicating that a relationship exists between these genes. However, when stratified by groups, hepatic expression of these downstream genes did not significantly change between obese type 2-diabetic and obese non-diabetic participants (ESM Fig. 1k,l), except for *PDK1*, which was increased in type 2 diabetes (ESM Fig. 1l). Taken together, hepatic *IRS2* is downregulated in the liver in those with diabetes and fatty liver, in comparison with the liver of obese non-diabetic participants. Importantly, our cohort showed negligible impact of confounding factors such as age, sex and BMI on *IRS2* gene expression, as proven by linear regression models (Fig. 1) supporting our hypothesis that aberrant *IRS2* expression might cause a diabetic phenotype independently of obesity.

### High variability of hepatic *IRS2* DNA methylation within transcription-factor binding motifs

**Fig. 2:**
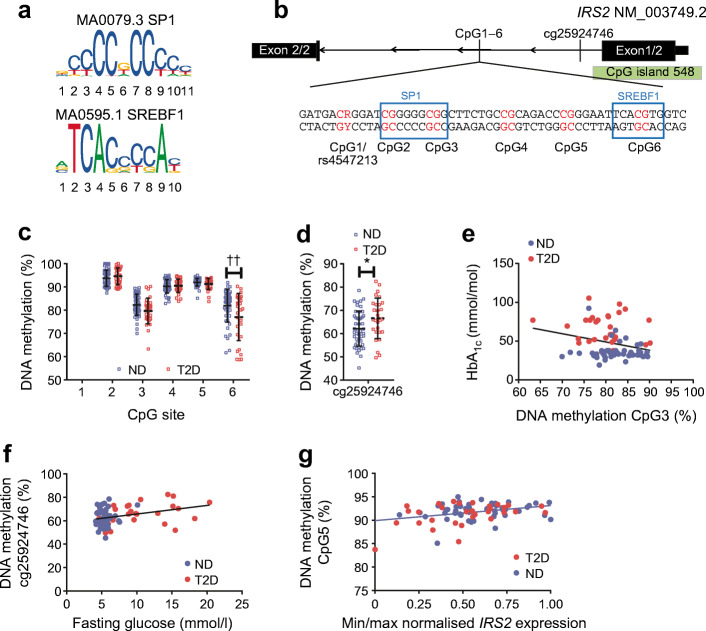
DNA-methylation pattern and sequence snippet of intron 1 of *IRS2.* (**a**) Transcription-factor binding profiles of SP1 (GC-box) and SREBF1 (E-box) from JASPAR database [14]. (**b**) An overview of the *IRS2* gene (NCBI reference sequence: NM_003749.2) and the location of the seven CpG sites analysed. The promoter-associated CpG island is shown in green. A sequence snippet from *IRS2* intron 1, with the location of CpG1–CpG6 within a GC-box (SP1 binding) and E-box (SREBF1 binding) motif, is magnified. (**c**) The DNA-methylation pattern of CpG2–CpG6 after stratification by diabetes status (non-diabetic [ND], *n* = 50; type 2 diabetic [T2D], *n* = 31). CpG6 within the E-box motif shows a difference in DNA methylation of 4.92% between T2D and ND (linear regression analysis: *p* = 0.0152; *p*_a_ = 0.0029). Each data point represents one individual; the black horizontal lines indicate the mean ± SD. (**d**) The graph shows per cent hepatic DNA methylation at cg25924746 (CpG1; affected by rs4547213) in T2D and ND obese participants (ND, *n* = 44; T2D, *n* = 28). Methylation was significantly higher in T2D participants (mean difference, 4.83%; *p* = 0.0208). This finding was sex-dependent and diminished after adjustment for intracohort variability (*p*_a_ = 0.0771). In (**c**) and (**d**), individual data points are shown with the mean (black horizontal line). (**e**) DNA methylation at CpG3 (GC-box) correlates negatively with HbA_1c_ values (*r* = −0.2674; *p*_a_ = 0.0281; *q* = 0.103; *n* = 81). (**f**) DNA methylation at cg25924746 correlates positively with fasting glucose (*r* = 0.3325, *p* = 0.0043; *n* = 72). This finding was sex-dependent and diminished after adjustment for intracohort variability, and was not significant after correction for multiple testing (*p*_a_ = 0.0600; *q* = 0.3555). (**g**) DNA methylation of CpG5 correlates with *IRS2* expression in the liver in the entire cohort (*r* = 0.3415, *p*_a_ = 0.0025; *q* = 0.0283; *n* = 81). Pearson’s correlation (*p*) and linear regression analysis (*p*_a_) were used to calculate the association between DNA methylation and the investigated parameters. Max, maximum; min, minimum. *p < 0.05; ††*p*_a_ < 0.01

Because hepatic *IRS2* expression is decreased in individuals with type 2 diabetes, we hypothesised that epigenetic mechanisms could play a role in this downregulation. Therefore, we performed in silico analysis for transcription-factor binding motifs containing a CpG dinucleotide (Fig. 2a). Two transcription-factor motifs in proximity to each other (recognition sites for SP1 and SREBF1) were found in *IRS2* intron 1 (Fig. 2b). Both transcription-factor binding motifs contain a central CpG site. Thus, we analysed DNA methylation of six CpG sites (CpG1–6) that span these SP1 and SREBF1 motifs in the liver of the obese non-diabetic and type 2 diabetic cohorts. Methylation of CpG6, which is centred in the SREBF1 E-box motif (Fig. 2b), was significantly decreased in the liver of individuals with type 2 diabetes (−4.92%; *p* = 0.0152; *p*_a_ = 0.0029; Fig. 2c). Additionally, we measured methylation at the *IRS2* promoter-associated CpG site cg25924746 (Fig. 2b), which was recently associated with altered fasting insulin and fasting glucose in a blood-based epigenome-wide study [12]. DNA methylation of cg25924746 is sex-dependent (*p* = 0.0004 for test between sex; data not shown), and was found to increase in the liver of obese diabetic participants, as compared with non-diabetic participants (4.83%; *p* = 0.0208; Fig. 2d), and correlated with fasting glucose (*p*_a_ = 0.0600; *q* = 0.0652; Fig. 2f). CpG3 methylation, which is located in the SP1 GC-box motif (Fig. 2b), correlated significantly with HbA_1c_ (*p*_a_ = 0.0281; *q* = 0.103; Fig. 2e). Importantly, DNA methylation at CpG1–6 within intron 1 of *IRS2* did not correlate with age, sex or BMI (data not shown).

Additionally, we assessed correlation of DNA methylation at the seven investigated CpG sites individually with *IRS2* mRNA levels (Fig. 2g and ESM Fig. 2a-e [data for CpG1 not shown]). DNA methylation of CpG5 correlated with *IRS2* expression in the liver of the entire cohort (Fig. 2g; *r* = 0.3415, *p*_a_ = 0.0025; *q* = 0.0283; *n* = 81). DNA methylation of the other investigated CpG sites did not correlate with *IRS2* gene expression after adjustment for age, sex and BMI, or after stratification for sex (data not shown), indicating that *IRS2* DNA methylation may only have a subtle influence on *IRS2* expression.

The SNP rs4547213 (G>A; also referred to as cg12195446, for example, in Illumina Infinium bead chip assay) affects the guanine of CpG1 [17]. Thus, DNA methylation at CpG1 is altered according to the genotype at this position and erased when individuals are homozygous carriers of the A allele (CpA-site; ESM Fig. 2f). Stratification for genotype did not result in significantly different DNA methylation at CpG1 (ESM Fig. 2f) between non-diabetic and type 2-diabetic obese participants. The G allele was numerically more frequent in individuals with type 2 diabetes in our cohort, but a χ^2^ test and regression analysis did not indicate a significant association between SNP rs4547213 and type 2 diabetes (ESM Fig. 2g). Surprisingly, the genotype at this SNP was significantly associated with DNA methylation at CpG3 (*p* = 0.0234; *p*_a_ = 0.0348; ESM Fig. 2h) as the G allele was associated with lower CpG3 methylation in obese type 2 diabetic participants, as compared with those without diabetes (*p* = 0.0350; ESM Fig. 2i). An eQTL analysis, including age, sex and BMI as cofactors, showed that the genotype at rs4547213 has no effect on *IRS2* gene expression, per se (*p* > 0.05; ESM Fig. 2j).

### *IRS2* DNA methylation alters reporter gene expression

**Fig. 3:**
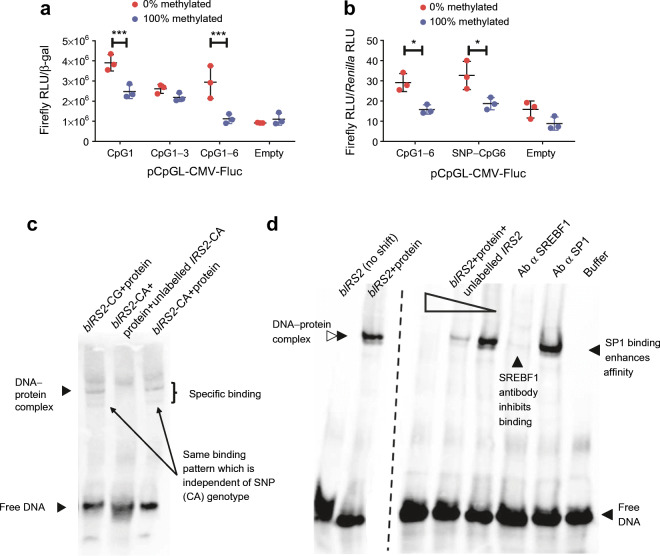
Methylation-sensitive luciferase reporter gene assay and EMSA using HepG2 cells to prove mechanistic influence of CpG regions analysed and validation of specific binding of transcription factors. (**a**) A methylation-sensitive luciferase reporter gene assay shows significant upregulation of the Firefly luciferase gene for unmethylated plasmid DNA (*n* = 3). These results indicate that methylation solely at CpG1 or CpG1–CpG6 influences the CMV promoter of the Firefly-luciferase-encoding gene. (**b**) The methylation- and polymorphism-sensitive luciferase reporter gene assay shows no difference between luciferase expression with the SNP rs4547213 genotype (SNP–CpG6), in which CpG1 is deleted, and cells containing the full CpG-site 1 (CpG1–CpG6) or the SNP (SNP–CpG6) (*n* = 3). (**c**) Biotinylated DNA (b*IRS2*) oligonucleotides containing either the CG (lane 1) or CA (lanes 2 and 3) genotypes were incubated with HepG2 nuclear extracts (protein) for EMSA. The EMSA shows a shift of biotinylated DNA–protein complexes, meaning that protein was bound. In lane 2, the reaction was additionally incubated with excess unlabelled DNA (unlabelled *IRS2*) which competes with the b*IRS2* and specifically erases the shift of labelled DNA. There is no observable difference in protein binding nor specificity between CG and CA genotypes (lane 1 and lane 3), therefore, protein binding is not influenced by the polymorphism rs4547213. (**d**) EMSA shows a shift of b*IRS2*, containing CpG1–CpG6, after incubation with nuclear extracts of HepG2 cells (b*IRS2* + protein, lane 2), which can be erased by incubation with different concentrations of unlabelled *IRS2* (12.5 pmol, 1.25 pmol, and 0.125 pmol, as indicated as gradient shape across lanes 3–5). Incubation of the binding reaction with an antibody against SREBF1 (Ab α SREBF1, lane 6) appeared to inhibit SREBF1 binding, reducing the intensity of the DNA–protein complex band, whereas incubation with an antibody against SP1 (Ab α SP1, lane 7) increased SP1 binding and, hence, the intensity of this band. As a control, the nuclear extraction buffer (Buffer, lane 8) shows no background signal. The dotted line indicates omission of three lanes (see ESM Fig. 3 for the unmodified blot). Data are shown as mean ± SEM. β-gal, β-galactosidase; RLU, relative luminescence units. *p < 0.05,***p < 0.001, repeated two-sided Student’s *t* test

To test if DNA methylation at the six transcription factor-spanning CpG sites investigated within intron 1 of *IRS2* has a functional effect on gene expression, we performed a methylation-sensitive reporter gene assay [15]. DNA methylation reduces luciferase expression by about 50–75% when it is solely of CpG1 or the sequence spanning CpG1 to CpG6 and, therefore, the SP1/SREBF1 motifs were included in the reporter construct (Fig. 3a). The genotype at SNP rs4547213, in which CpG1 is deleted, had no direct influence on luciferase expression, as tested by comparing the 0% methylated inserts containing vectors harbouring the SNP with vectors containing the full CpG site 1 (Fig. 3b). Thus, the six CpGs identified that span the two transcription-factor binding sites appear to have a methylation-dependent regulatory effect on promoter activity.

### Specific SP1 and SREBF1 binding to *IRS2*

According to public data the regions of *IRS2* investigated here lie in a DNaseI hypersensitivity cluster [18], but transcription factor chromatin immunoprecipitation sequencing (ChIP-seq) peak data (Encyclopedia of DNA Elements [ENCODE3] project; [19, 20]) indicate no transcription factor binding in HepG2 cells or the liver. For confirmation of our in silico-proposed transcription-factor binding motifs, we performed an EMSA to test whether nuclear protein would bind to our CpG sites of interest. Biotin-labelled oligonucleotides containing the six CpG sites (CpG1–6) were shifted, indicating that protein was bound (Fig. 3d). Incubation with nuclear extract from HepG2 cells led to a shift of labelled oligonucleotides (Fig. 3c,d). This shift was not influenced by the polymorphism rs4547213 (Fig. 3c). Incubation with different concentrations of unlabelled oligonucleotide led to a reduction of bound labelled oligonucleotide and, therefore, indicates specificity of binding (Fig. 3d). Incubation of nuclear extract with an antibody against SREBF1 reduced oligonucleotide-binding affinity, indicating that SREBF1 can no longer bind the oligonucleotide when it is occupied by an antibody (Fig. 3d), whereas the SP1 antibody seemingly enhanced binding affinity. These results could indicate specific binding of the transcription factors SREBF1 and SP1 at their predicted motifs within intron 1 of *IRS2*.

### Hepatic miRNA let-7e-5p is increased in type 2 diabetic participants

**Fig. 4:**
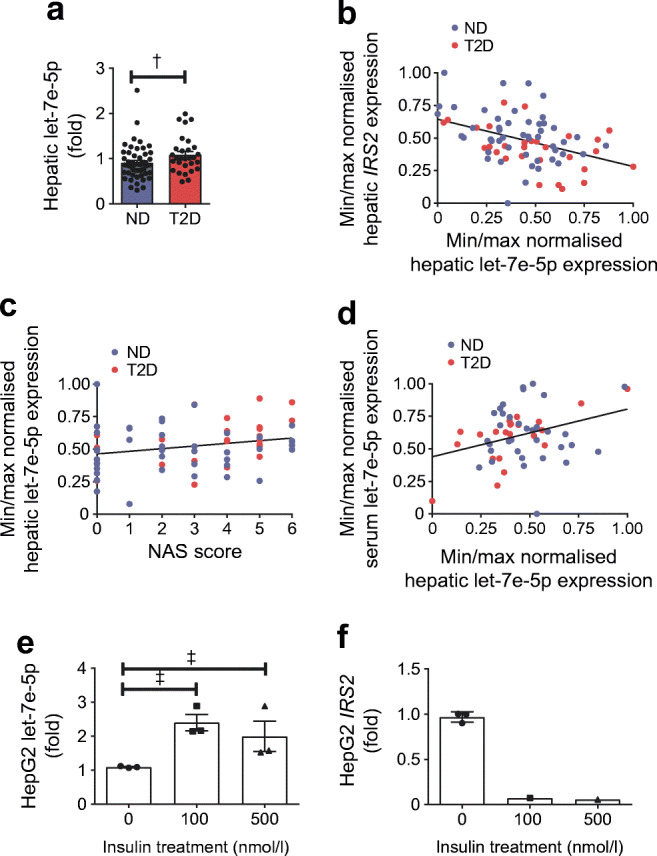
Altered hepatic expression of miRNA let-7e-5p in type 2 diabetic (T2D) and non-diabetic (ND) participants. (**a**) Hepatic let-7e-5p expression is increased 1.2-fold in obese T2D individuals (*p*_a_ = 0.0450; ND, *n* = 49; T2D, *n* = 29). (**b**) Hepatic expression of *IRS2* correlates negatively with let-7e-5p expression (*r* = −0.4133, *p* = 1.7 × 10^−4^; *q* = 0.0019; *n* = 78). (**c**, **d**) Hepatic let-7e-5p expression does not correlate with liver steatosis and fibrosis status (NAS) (**c**; *r* = 0.2476, *p*_a_ = 0.0479; q = 0.2123; *n* = 67) or serum let-7e-5p expression (**d**; *r* = 0.3284, *p* = 0.0135; *q* = 0.2123; *n* = 56). (**e**) Intracellular expression of let-7e-5p is induced in HepG2 cells after treatment with insulin (100 nmol/l and 500 nmol/l) in high-glucose medium (25 mmol/l) for 24 h analysed by one-way ANOVA (*p* = 0.0279, overall difference between the means), with post-hoc test and adjustment for multiple testing by the FDR method (^‡^*q* < 0.05, 0 nmol/l vs 100 nmol/l or 0 nmol/l vs 500 nmol/l); *n* = 3. (**f**) Expression of *IRS2* in HepG2 cells after treatment with insulin (100 nmol/l and 500 nmol/l; *n* = 3) in high-glucose medium (25 mmol/l) for 24 h is only detectable after insulin treatment for one experiment, therefore, no statistical analysis could be performed. MiRNA expression was normalised to the expression of a housekeeper miRNA (for liver and HepG2 cells) or to a spike-in control (for serum). In (**a**), (**e**) and (**f**), miRNA expression is shown as mean fold ±SEM relative to ND (**a**) or 0 nmol/l insulin (**e**, **f**), as calculated by the ${2}^{-{\Delta  \Delta  C}_t}$ method. In (**c**) and (**d**), Pearson’s correlation or Spearman’s correlation (*p*) and linear regression analysis (*p*_a_) was used to calculate an association between gene expression (ΔC_t_) and other parameters. For visualisation, miRNA expression was normalised by minimum (min)/maximum (max) normalisation (1 − (x_i_ − x_min_)/(x_max_ − x_min_)) to a scale from 0 (min expression) to 1 (max expression), whereby *x*_i_ is the current ΔCt value of a specific gene, *x*_min_ is the minimum ΔCt value of all measurements of a specific gene and *x*_max_ is the maximum ΔCt value of all measurements of a specific gene. Gene expression is shown as normalised expression on the axes. †*p*_a_ < 0.05; ^‡^*q* < 0.05

Alongside DNA methylation, miRNAs represent another important epigenetic mechanism that regulates gene expression. Because epigenetic mechanisms often act synergistically, we performed data mining to identify miRNAs that possibly co-regulate *IRS2* expression together with DNA methylation. Experimentally validated databases (DIsplacement ANAlyzer [DIANA] tools, TarBase v.8 [21]), as well at databases with predicted interactions (TargetScan Release 7.2 [22]), predict a conserved 8-mer site of let-7e-5p within the 3′ untranslated region (UTR) of *IRS2*. Hepatic let-7e-5p was increased 1.2 ± 0.08-fold in individuals with type 2 diabetes (*n* = 29), as compared with non-diabetic obese participants (*n* = 49) (*p* = 0.0332; *p*_a_ = 0.0450; Fig. 4a), and a negative correlation between let-7e-5p and *IRS2* expression was found to exist (*r* = −0.4133, *p* = 1.7 × 10^−4^; *q* = 0.0019; Fig. 4b). Moreover, let-7e-5p expression was weakly correlated with NAS (*r* = 0.2476; *p*_a_ = 0.0479; *q* = 0.2123; Fig. 4c); this correlation was not significant after correction for multiple testing (ESM Table 4). Hepatic let-7e-5p expression did not correlate with age, sex or BMI (data not shown). To test whether let-7e-5p could be a candidate biomarker for type 2 diabetes in easily accessible tissues or body fluids, we measured let-7e-5p expression in non-haemolytic serum of *n* = 56 participants. We did not find differences in serum let-7e-5p expression between type 2 diabetic and non-diabetic participants, and it correlated only weakly and not significantly with hepatic let-7e-5p expression after correction for multiple testing (*r* = 0.3284, *p* = 0.0135; *q* = 0.2123; Fig. 4d, ESM Table 4).

Finally, we were interested to determine whether hepatic miRNA expression could be induced by hyperinsulinaemia and hyperglycaemia. We treated HepG2 cells with either 100 nmol/l or 500 nmol/l insulin in high-glucose medium, or with no insulin in low-glucose conditions as a control. HepG2 let-7e-5p expression was increased by acute hyperinsulinaemic and hyperglycaemic conditions as compared with the control (100 nmol/l insulin: 2.4-fold; 500 nmol/l insulin: 2-fold; Fig. 4e). Concomitantly, gene expression of *IRS2* was decreased and below the limit of detection in two out of three biological replicates in insulin-treated HepG2 cells (Fig. 4f). Because *IRS2* expression after insulin treatment was only detectable in one of the three biological replicates, no statistical analysis could be performed.

## Discussion

IRS2 is an essential mediator of insulin signalling [2]. Here, we show a multi-layered epigenetic mechanism that could be involved in downregulation of *IRS2* expression in the liver of obese individuals with type 2 diabetes, as compared with obese individuals without type 2 diabetes. It is suggested that this regulation is facilitated by a combination of two epigenetic mechanisms, DNA methylation at SREBF1 and SP1 binding sites, and upregulation of hepatic miRNA let-7e-5p. DNA methylation of the *IRS2* promoter-associated EWAS marker cg25924746 is increased (in a sex-dependent manner) in the liver of type 2 diabetic participants, further strengthening our hypothesis that hepatic DNA methylation of *IRS2* could play a role in type 2 diabetes. Additionally, the genotype at the intronic SNP rs4547213 is associated with DNA methylation at the SP1 binding site, which may, in part, explain why this SNP was associated with traits of glucose metabolism in a recent large-scale genome-wide association study (GWAS) [23].

Basal regulation of *IRS2* gene expression by transcription factors is well characterised*.* Forkhead transcription factors, such as forkhead box O (FOXO)1/3, bind to the insulin responsive element (IRE) of the *IRS2* promoter to increase *IRS2* expression during insulin stimulation, leading to an activation of the phosphatidylinositol 3-kinase (PI3K)/Akt1/2 signalling pathway [24]. The IRE of *IRS2* overlaps with a sterol regulatory element (SRE), leading to competition between FOXO transcription factors and SREBF1a/1c, acting as a regulatory repressor of *IRS2* [25]. Here, we propose additional regulators of hepatic *IRS2* expression that might function as fine-tuning mechanisms depending on glycaemic state. One of the proposed transcription factors involved in this regulation is SREBF1. This factor is particularly interesting because it was previously shown to act as a translational repressor on *IRS2* [25]*.* SREBF1c, the main SREBF1-isoform in the liver, is known to be involved in de novo lipogenesis by activating lipogenic genes, including fatty acid synthase (FASN) or stearyl CoA desaturase (SCD) [26, 27], which were found to be increased, although non-significantly, in our cohort (ESM Fig. 1k), suggesting that SREBF1c is activated in our sample set. Consequently, through activation of *SREBF1* and let-7e-5p, and the subsequent repression of *IRS2* expression, the liver may be epigenetically arrested in a state of active lipogenesis.

SP1 is an important cofactor for the binding of SREBF1 to DNA [28]. Recently it was shown that DNA binding of SP1 [29, 30] and SREBF1 [31] is hindered by DNA methylation. In our study, methylation within the SREBF1 E-box motif was significantly decreased in individuals with type 2 diabetes, as compared with those without, despite receiving glucose-lowering medication. Enhanced binding of SREBF1 due to DNA-hypomethylation might, therefore, be a mechanism that cannot be reversed by glucose-lowering medication and, instead, is putatively influenced by overall glycaemic control which is not as good in the type 2 diabetic group as compared with the non-diabetic group.

Because gene transcription can be regulated by DNA methylation of CpG islands in gene promoters, we studied DNA methylation of cg25924746. This CpG site is located in a CpG-island shore, which are generally known to have variable DNA methylation [32, 33]. In our cohort, we reproduced the finding that DNA methylation of cg25924746 is altered in type 2 diabetes [12]. We found increased DNA methylation in the type 2 diabetic obese participants, as compared with non-diabetic obese subjects. However, correlations between cg25924746 and clinical parameters of glucose homeostasis were only significant in a sex-dependent manner, which became insignificant when adjusting for sex and for multiple testing. This discrepancy between our findings and previously published data could be due to the smaller sample size in our study and the fact that we lacked a lean control group. Furthermore, we investigated DNA methylation of cg25924746 in liver tissue, while it was previously only studied in DNA derived from leucocytes [12]. Therefore, caution should be applied when comparing DNA methylation across tissues [34].

The genotype at the neighbouring SNP rs4547213 correlates with DNA methylation at the CpG3/SP1 motif. According to data in the GWAS central database [35], the polymorphism itself has been shown to be associated with fasting plasma glucose (*p* = 0.010; as determined by the Meta-Analyses of Glucose and Insulin-related traits Consortium [MAGIC], which combined 21 GWAS) [36]. In addition, a second GWAS meta-analysis [23], which included more than 800,000 individuals of European ancestry from 32 GWAS, found an association between rs4547213 and the incidence of type 2 diabetes (*p* = 6.8 × 10^−7^). In our cohort, we could not reproduce the significant association between rs4547213 and type 2 diabetes, which is likely to be due to our smaller study cohort. Our mechanistic data from the luciferase reporter gene assay and the eQTL analysis did not show any effect of the polymorphism on gene expression per se. Nevertheless, type 2 diabetic participants with preserved DNA methylation due to the G allele at CpG1, and not the putatively protective A allele, showed decreased DNA methylation at the CpG3/SP1 motif in comparison with participants with type 2 diabetes and the AA genotype (ESM Fig. 2i). This could, in theory, lead to enhanced SP1/SREBF1 binding and downregulation of *IRS2* expression.

The findings from the luciferase reporter gene assay applied in this study indicates that higher methylation will result in decreased promoter activity. Our data derived from liver biopsies indicate a regulation in the opposite direction. It should be noted that the luciferase assay was used to analyse the effect of DNA methylation on a CMV promoter in an artificial cell-culture system and effects of competing transcription factors, as already described between SREBF1 and FOXO1/3, are not physiologically resembled. Nevertheless, we prove a mechanistic effect of DNA methylation on gene expression and the specificity of SREBF1 and SP1 binding, although our method was not sensitive enough to observe direct methylation-dependent binding of both proteins.

Furthermore, hepatic let-7e-5p negatively correlated with *IRS2* expression in our cohort. Specific targeting of let-7e-5p to the 3′UTR of *IRS2* has previously been established and has been specifically shown in the liver [8, 9, 37, 38]. We, therefore, believe that let-7e-5p influences *IRS2* expression despite not finding correlations between hepatic let-7e-5p and clinical parameters after correction for multiple testing in our cohort. Moreover, high insulin and glucose treatment was able to acutely stimulate let-7e-5p expression in HepG2 cells leading to *IRS2* downregulation. A previous study on miRNAs of the let-7 family already pointed towards a function of let-7e-5p upregulation in disease progression of hepatocellular carcinoma (HCC), which is often a consequence of NAFLD [39]. Therefore, in the context of hepatic insulin resistance and fat accumulation, let-7e-5p could be an attractive target for miRNA inhibitor treatments, as seen for miRNA-122 in hepatitis C infection [40, 41].

### Conclusion

**Fig. 5:**
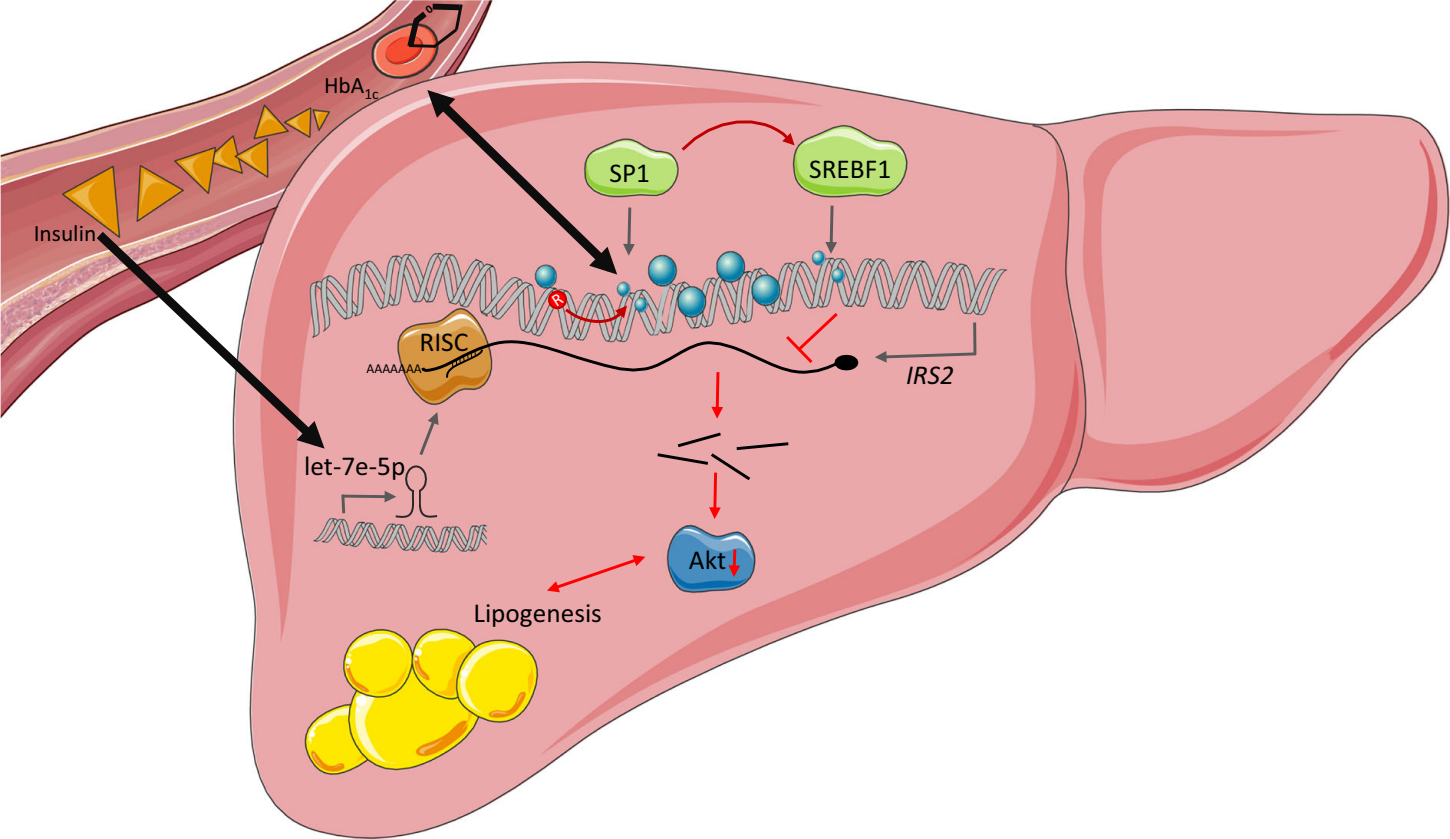
Proposed multi-layered network participating in *IRS2* dysregulation in type 2 diabetes. DNA methylation at SP1- and SREBF1-binding sites, together with elevated miRNA let-7e-5p levels, are associated with decreased *IRS2* transcription. Elevated let-7e-5p miRNA levels might result from increased serum insulin concentrations (single-ended thick black arrow) and might cause degradation of *IRS2* mRNA (as illustrated by the short black lines above Akt), for example, via the RNA-induced silencing complex (RISC). A decrease in DNA methylation at the putative SP1 binding site is associated with increased HbA_1c_ levels (double-ended thick black arrow) and the genotype at rs4547213 (R, red arrow). This might lead to compromised insulin action, resulting in reduced Akt signal transmission and increased gluconeogenesis and lipogenesis, further fuelling liver steatosis (indicated as lipogenesis) and hepatic insulin resistance. This figure was created using images from Servier Medical Art (http://smart.servier.com). Servier Medical Art by Servier is licensed under a Creative Commons Attribution 3.0 Unported License

In conclusion, we identified a complex network of epigenetic DNA methylation, miRNA and genotypes that could be involved in the regulation of *IRS2* expression in the liver of diabetic individuals (Fig. 5). Within this network, our data suggest that let-7e-5p might play a larger role than DNA methylation in modulating hepatic *IRS2* expression. As the targeting of miRNAs in liver is already used for human therapies [40, 41], inhibition of hepatic let-7e-5p might be a novel therapeutic target to support the treatment of hepatic insulin resistance and fatty liver.

## Electronic supplementary material

ESM (PDF 935 kb)

## Data Availability

The data that support the findings of this study are available from the corresponding author upon reasonable request.

## Abbreviations

ALT: Alanine aminotransferase
CMV: Cytomegalovirus
EMSA: Electrophoretic mobility shift assay
eQTL: Expression quantitative trait loci
EWAS: Epigenome-wide association study
FDR: False discovery rate
FOXO: Forkhead box O
GWAS: Genome-wide association studies
IRE: Insulin responsive element
MiRNA: MicroRNA
NAFLD: Non-alcoholic fatty liver disease
NAS: Non-alcoholic fatty liver disease activity score
SP1: Specificity-protein-1
SREBF1: Sterol regulatory element binding transcription factor 1
UTR: Untranslated region
