# Supplementary material for: Multi-layered epigenetic regulation of IRS2 expression in the liver of obese individuals with type 2 diabetes
Source: Diabetologia. 2020 Jul 24;63(10):2182–93. doi: 10.1007/s00125-020-05212-6 (PMC7476982; doi:10.1007/s00125-020-05212-6)
Supplement: Supplementary file 1 — (PDF 935 kb) [file 125_2020_5212_MOESM1_ESM.pdf]

Electronic supplementary material (ESM)

Krause et al. Multi-layered epigenetic regulation of *IRS2* expression in the liver of obese individuals with type 2 diabetes

ESM methods

1 Study design and patients

Briefly, all patients undergoing bariatric surgery were asked to participate in tissue collection. As a consequence, samples were not matched. Blood samples for serum extraction were drawn at the day of surgery after an over-night fast. All participants signed informed consent.

2 DNA methylation measurement

DNA was extracted from 25 mg of snap frozen liver using the QIAmp mini kit (QIAGEN, Hilden, Germany) as indicated by the manufacturer with minor changes in volume of added PBS (80 µl) and buffer ALT (180 µl) for homogenization. After homogenization (*Bead Ruptor Omni International)* and addition of protease K, the homogenate was stored at room temperature for 11-14 days prior final extraction steps, which is in accordance with the instructions of the manufacturer (Qiagen) and DNA methylation was shown to be stable under such storage conditions [1, 2].

All primer sets, including an assay for the analysis of CpG1 to CpG5, a reverse assay for CpG6 and a third primer assay for cg25924746, were designed using the PyroMark Assay Design 2.0 software. Bisulphite PCR was performed with the PyroMark PCR kit with 15 to 20 ng of input bisDNA for 40 cycles. Bisulphite-treated control DNA with known methylation quantities (EpiTect control DNA, QIAGEN, Hilden, Germany) was used for assay establishment and validation. Furthermore, systematic quality controls were performed before pyrosequencing including tests for primer-dimers and primer self-annealing. These quality controls included sequencing of i) reverse and sequencing primer w/o PCR product, ii) reverse primer only, iii) sequencing primer only, and iv) PCR product w/o primers. The inter-assay variance is below 3% and the intra-assay variance is below 4% for all assays. DNA methylation was measured by bisulphite pyrosequencing using the PyroMark Q48 and PyroMark Q48 Advanced reagents (QIAGEN, Hilden, Germany) according to protocol.

3 RNA isolation and gene expression analysis

Total RNA was extracted from 25 mg of snap frozen liver using the Mirneasy mini kit (QIAGEN, Hilden, Germany) and quantified spectrometrically. 2 µg of RNA was reverse transcribed into cDNA using the SuperScript VILO cDNA synthesis kit (Invitrogen, Carlsbad, US).

31 potential housekeeping genes were tested with the TaqMan Array Human Controls Plate (Applied Biosystems, Foster City, US). According to the NormFinder algorithm [3] *CASC3* (Hs00201226_m1, Applied Biosystems, Foster City, US) was the best housekeeping gene and therefore all expression data were normalised to *CASC3* expression using the ΔΔCt method. The following TaqMan gene expression assays were used: *IRS2* (Hs00275843_s1); *PCK1* (Hs00159918_m1); *G6PC* (Hs00609178_m1); *PDK1* (Hs01561847_m1); *ACACA* (Hs01046047_m1); *ACACB* (Hs00153715_m1); *FASN* (Hs01005622_m1); *SCD* (Hs01682761 m1); *PNPLA2* (Hs00386101_m1); *FOXO1* (Hs00231106_m1); *ELOVL6* (Hs04260088_m1); *CASC3* (Hs00201226_m1). Each qPCR reaction contained 10 ng of mRNA-cDNA.

For hepatic miRNA cDNA synthesis, 10 ng of total RNA was reverse transcribed with the TaqMan Advanced cDNA synthesis kit (Applied Biosystems, Foster City, US). Hsa-let-7e-5p expression (478579_mir, Applied Biosystems, Foster City, US) was measured by TaqMan Advanced miRNA assay and calculated using the ΔΔCt method with hsa-miR-24-3p (477992_mir, Applied Biosystems, Foster City, US) as housekeeping gene.

4 Measurement of serum miRNA

Serum miRNA was extracted from 200 µl frozen serum with the miRNeasy Serum/Plasma Advanced kit (QIAGEN, Hilden, Germany) using the recommended spike-in control cel-miR-39-3p (QIAGEN, Hilden, Germany). Serum miRNAs were reverse transcribed with the qScript microRNA cDNA synthesis kit (QuantaBio, Beverly, US) using 5 µl eluate (corresponding to 40 µl serum input) for cDNA synthesis in 20 µl cDNA reaction mixture. Gene expression was measured by qPCR using the FastStart Universal SYBR Green Master (Roche, Basel, CH), self-designed qPCR primer (ESM Table 2) and universal primer from the qScript kit. Each qPCR reaction contained 266 nl input serum and target expression was normalized to the spike-in control cel-miR-39-3p expression using the ΔΔCt method.

5 Methylation-sensitive luciferase reporter gene assay

Luciferase-plasmids with a CpG free backbone and CpG-free CMV promoter (pCpGL-CMV-Fluc [4]) where created with different inserts (ESMTable 2). Subsequent restriction digestion was performed with *HindIII* and *NcoI* (NEB, Ipswich, US) and ligation with double stranded oligonucleotides described above by Quick ligation protocol (M2200, NEB, Ipswich, US). Ligation mix was transformed into One Shot PIR1 chemically competent cells (Invitrogen, Carlsbad, US) with zeocin resistance. Plasmids were extracted by QIAprep Spin Miniprep kit (QIAGEN, Hilden, Germany). Consequently, plasmids were either *in vitro* methylated using *SssI* methylase (NEB, Ipswich, USA) or mock methylated (incubation without *SssI*). HepG2 ([HEPG2] (ATCC® HB8065™)) cells were purchased from ATCC (Manassas, US) and are regularly checked for mycoplasma in a standardised manner by qPCR test for mycoplasma performed under ISO17025 accreditation to ensure work with negatively tested cells. HepG2 cells were plated into 96-well plates one day prior transfection. At 60 – 70% confluency cells were co-transfected as triplicates with 100 ng luciferase reporter plasmid and either 500 ng β-galactosidase control plasmid (pCMV-bGal) or 10 ng SV40 renilla control plasmid (pRL-SV40)

per well using Lipofectamin 3000 (Invitrogen, Carlsbad, US). Cells were harvested after 24 h of incubation in 50 µl passive lysis buffer per well and luciferase activity was measured in 20 µl lysate by DualGlo kit (Promega, Madison, US). For β-galactosidase normalization, β-galactosidase assay was performed by β Galactosidase Enzyme Assay System (Promega, Madison, US). All luciferase assays were performed three times with three technical replicates each and the mean of all three experiments is shown.

6 Electrophoretic mobility shift assay

20 µl of binding reaction containing 10 mM Tris, 50 mM KCl, 1 mM DTT, 50 ng Poly dI dC, 10 µM $ZnSO_4$, 0.2 ug BSA, 6.5 µg HepG2 nuclear extract (NE-PER Nuclear and Cytoplasmatic Extraction Reagents, Thermo Fisher) and 50 fmol biotinylated oligo (referred to as *labelled DNA*) were incubated for 30 min on ice prior gel loading on 6 % (wt/vol.) DNA retardation gel (Invitrogen, Carlsbad, US). For supershift assays, nuclear extract was incubated with a specific antibody (polyclonal IgG α SREBF1 PA1-46142, polyclonal IgG α Sp1 PA5-29165, both Invitrogen Antibodies, Carlsbad, US) for one hour on ice before gel-loading. Protein-DNA complexes were plotted on nylon membranes and detected via chemiluminescence using the Chemiluminescent Nucleic Acid Detection Module (Thermo Fisher, Waltham, US). Specificity of protein binding was tested for by competition experiments in which gradients of un-biotinylated oligos (referred to as *unlabelled DNA*) were added to the incubation mixture prior to gel loading. The competitive erasure of protein binding to IRS2 was repeated five times. The incubation with SREBF1-antibody and SP-1 antibody was repeated three times. The effect of SNP rs4547213 on protein binding was performed twice. One representative blot is shown.

7 Insulin treatment of HepG2 cells

HepG2 cells (200.000 cells per ml) were plated in 6-wells prior insulin treatment. At 50 – 60 % confluency, cells were cultivated in high glucose (25 mmol/l) DMEM supplemented with 0.5 % (wt/vol.) BSA and insulin (100 nmol/l or 500 nmol/l). Cells cultivated in low glucose (8.3 mmol/l) DMEM supplemented with 0.5 % (wt/vol.) BSA served as control. After 24 h of treatment, cells were washed once with PBS and lysed in 700 µL QIAzol as indicated by Mirneasy mini kit (QIAGEN, Hilden, Germany). All subsequent miRNA-cDNA and mRNA-cDNA synthesis steps were performed as described above. Insulin treatment was performed three times in duplicates each and the mean of all experiments is shown.

8. General statistics

Prior statistical calculations, outliers were identified using the robust regression and outlier removal (ROUT) method of GraphPad Prism version 7 (GraphPad Software, La Jolla, US) applying Q = 1 %.

Relative gene expression was calculated by the $\Delta\Delta C_t$ method, using a respective housekeeper gene for normalisation. Expression data was normalised to the respective control group. Results were either visualised as mean fold +/- SEM as group overview or normalised by minimum(min)/maximum (max) normalisation $(1 - (x_i - x_{min})/(x_{max} - x_{min}))$ to a scale from 0

(minimum expression) to 1 (maximum expression) for correlation analysis. Thereby x_i is the current ΔC_t value of a specific gene, x_{min} is the minimum ΔC_t value of all measurements of a specific gene and x_{max} is the maximum ΔC_t value of all measurements of a specific gene.

DNA methylation is shown as individual % methylation values which were calculated by the PyroMark Q48 Advanced Software (QIAGEN, Hilden, Germany). For plotting, each data point represents one analysed individual. The black line indicates the mean±SD.

Regression analyses were performed using MATLAB R2018a by fitting a linear regression model of the form $y = \beta_0 + \beta_1 X_1 + \ldots + \beta_n X_n$ with additional predictors β_1 to β_n (The MathWorks, Natick, US). Logistic regression analyses were similar performed using a multinomial logistic regression. Age, sex and BMI were used as predictors to control for the intracohort variance. Thereby generated $p_a<0.05$ was assumed as significant.

Linear and rank correlation analyses were also performed by MATLAB R2018a using the corr function either as Pearson or as Spearman correlation. Two-sided Student's t test was used for the comparison of means for normally distributed measurements. Two-sided Wilcoxon rank sum test was used for nonparametric tests. One-way ANOVA was used to compare the means of more than two groups with a post hoc test to compare individual means. Post hoc-calculated p values were adjusted for multiple testing by the FDR method of Benjamini and Hochberg and a q<0.05 was assumed as significant (GraphPad Prism, version 7; GraphPad Software, La Jolla, CA, USA). Moreover, we tested separately the influence of age and BMI by either correlating DNA methylation or gene expression with both factors. For the influence of sex on DNA methylation or gene expression, we performed a group stratification between male and female individuals and calculated a two-sided Wilcoxon rank sum test. A p<0.05 was assumed significant. P values were adjusted for multiple comparison by using the p.adjust function of R version 3.5.1 (RStudio, Boston, US) and controlling of the false discovery rate (FDR) with the method of Benjamini and Hochberg. Therefore, we applied a FDR = 15 % setting the level of significance to q<0.15.

After controlling for Hardy-Weinberg equilibrium, a χ^2 test with $\alpha = 0.05$ was used to test for an association between the polymorphism and the incidence of type 2 diabetes. The effect of the polymorphism on *IRS2* gene expression was assessed by calculation of a quantitative trait loci (eQTL) analysis performed by a linear mixed effect model (lmer) in R. The genotype of the polymorphism was used for the random effect on gene expression. Age, sex and BMI were used as cofactors (fixed effects).

All p values are denoted in the figures as *p<0.05, **p<0.01,***p<0.001, †$p_a<0.05$, ††$p_a<0.01$, ‡q<0.05.

ESM Tables

ESM Table 1: Clinical characteristics of subjects, stratified by medical examination into subjects with type 2 diabetes (T2D) or without type 2 diabetes (non-diabetic, ND). Significant differences in the cohort are indicated by bold p values (p<0.05).

	ND			T2D			
	Mean	STD	n	Mean	STD	n	**p value**
HbA1c (%)	5.48	1.02	50	8.15	1.68	31	**2.79E-13**
HbA1c (mmol/mol)	36.44	11.12	50	65.52	18.36	31	**2.79E-13**
NAS (score)	2.28	2.09	43	3.64	1.92	25	**1.08E-02**
Sex	m=39	w=11	50	w=19	m=12	31	**2.12E-02**
BMI (kg/m^2)	53.93	11.39	50	51.35	9.27	31	2.99E-01
Age (y)	39.60	10.87	50	49.84	11.90	31	**1.86E-04**
Glucose (mmol/l)	6.11	0.63	48	9.97	4.35	30	**1.07E-08**
Insulin (pmol/l)	99.45	92.14	49	164.34	179.52	26	**4.49E-02**
Creatinine (µmol/l)	72.56	13.52	48	100.42	45.53	30	**1.94E-04**
total cholesterol (mmol/l)	4.88	0.83	47	5.03	1.39	29	5.63E-01
triglycerides (mmol/l)	2.12	1.24	47	3.03	1.48	29	**5.73E-03**
HDL (mmol/l)	1.19	0.32	47	1.08	0.34	29	1.57E-01
LDL (mmol/l)	2.75	0.81	45	2.49	1.04	25	2.48E-01
GGT (µkat/l)	0.68	0.58	48	1.29	1.40	30	**1.01E-02**
CRP (mg/l)	14.94	11.57	48	16.76	15.06	29	5.58E-01
TSH (mU/l)	2.17	1.13	45	3.04	7.98	30	4.79E-01
vitamin B-12 (pmol/l)	529.64	310.62	44	592.21	341.17	29	4.27E-01
folic acid (nmol/l)	13.99	8.79	44	20.90	12.74	29	**8.53E-03**
AST (nkat/l)	389.17	242.33	48	617.83	618.33	30	**2.64E-02**
ALT (nkat/l)	505.50	360.50	48	628.33	308.17	30	1.31E-01

ESM Table 2: List of Primers and oligonucleotides.

Name	Sequence	Use
**Oligonucleotides for luciferase reporter assay**		
pCpGL-CMV-RLuc- CpG1	5’-TGACGGGAT	Luciferase plasmid insert containing the CpG1 (cg12195446)
pCpGL-CMV-RLuc-SNP	5’-TGACAGGAT	Luciferase plasmid insert containing the SNP (rs4547213)
pCpGL-CMV-RLuc- CpG1-CpG3	5’-TGACGGGATCGGGGGCGGCTTC	Luciferase plasmid insert containing the CpG1 (cg12195446) and CpG2, CpG3
pCpGL-CMV_RLuc- CpG1_CpG6	5’-TGACGGGATCGGGGGCGGCTTCTGCCGCAGACCCGGGAATTCACGTGGTC	Luciferase plasmid insert containing the CpG1 (cg12195446) and CpG2, CpG3, CpG4, CpG5 and CpG6
pCpGL-CMV_RLuc- SNP_CpG6	5’-TGACAGGATCGGGGGCGGCTTCTGCCGCAGACCCGGGAATTCACGTGGTC	Luciferase plasmid insert containing the SNP (rs4547213) and CpG2, CpG3, CpG4, CpG5 and CpG6
**Oligonucleotides for EMSA**		
IRS2_EMSA_biotin	5’-biotin-ACGGGATCGGGGGCGGCTTCTGCCGCAGACCCGGGA ATTCACGTGGTC	Oligonucleotide for shift and binding assay (EMSA) containing CpG1 (cg12195446), CpG2, CpG3, CpG4, CpG5 and CpG6
IRS2_EMSA	5’-ACGGGATCGGGGGCGGCTTCTGCCGCAGACCCGGGA ATTCACGTGGTC	Competitive oligonucleotide for shift and binding assay (EMSA)
IRS2_EMSA_SNP	5’-biotin-ACAGGATCGGGGGCGGCTTCTGCCGCAGACCCGGGAATTCACGTGGTC	Oligonucleotide for shift and binding assay (EMSA) containing the SNP (rs4547213) instead of CpG1 (cg12195446), CpG2, CpG3, CpG4, CpG5 and CpG6
**Primer for bisulphite pyrosequencing**		
IRS2_ cg12195446 _r4547213 _CpG5_forward	5’-GTAGGGTAGTTATGGGTGAGG	Bisulphite pyrosequencing PCR for CpG1 - CpG5, annealing temperature 56 °C
IRS2_ cg12195446 _r4547213 _CpG5_reverse	5’-biotin-TCCCCAACAAAATTTAAATACCTCTA	Bisulphite pyrosequencing PCR for CpG1 - CpG5, annealing temperature 56 °C
IRS2_ cg12195446 _r4547213 _CpG5_sequencing	5’-GGGTGGTTATTGAGAT	Bisulphite pyrosequencing sequencing primer for CpG1 - CpG5
IRS2_CpG6_	5’-biotin-GGGGTTAAGAGGAAGTTTTTGAGTAGA	Bisulphite pyrosequencing PCR for CpG6, annealing temperature 54 °C

reverseAssay _forward		
IRS2_CpG6_ reverseAssay _reverse	5’-CACCACCTCCTAACCAATCT	Bisulphite pyrosequencing PCR for CpG6, annealing temperature 54 °C
IRS2_CpG6_ reverseAssay _sequencing	5’-GGAAGGGAGTTTTTTTATTTTTAA	Bisulphite pyrosequencing sequencing primer for CpG6
IRS2_cg25924746 _forward	5'-GATTAAGGTGGGTTGGAGAT	Bisulphite pyrosequencing PCR for cg25924746
IRS2_cg25924746 _reverse	5'-AAATTCACCCCACAACTATAATTTTA	Bisulphite pyrosequencing PCR for cg25924746
IRS2_cg25924746 _sequencing	5'-GTATTTGTTTTATAGTATATTTAGG	Bisulphite pyrosequencing sequencing primer for cg25924746
IRS2_genotype _rs4547213 _forward	5'-GGGCAGTCATGGGTGAGG	PCR for genotyping of rs4547213 by pyrosequencing
IRS2_genotype _rs4547213 _reverse	5'-biotin-GGGAAGAGCGTGCACCTAC	PCR for genotyping of rs4547213 by pyrosequencing
IRS2_genotype _rs4547213 _sequencing	5'-TGGCCACTGAGATGA	Genotyping of rs4547213 by pyrosequencing
qPCR primer for microRNA analysis		
hsa-let-7e-5p _forward	5'-GCAGTGAGGTAGGAGGTTG	qPCR miRNA Serum
cel-miR-39-3p _forward	5'-CACCGGGTGTAAATCAGCTTG	qPCR miRNA Serum

Relevant CpGs (and CpA for SNP) dinucleotides are underlined.

ESM Table 3. p-values of all correlation analyses after adjustment for age, sex and BMI

**P-values adjusted for age, sex and BMI; p < 0.05**									
	**let-7e-5p liver**	**IRS2 liver**	**let7e-5p serum**	**cg25924746**	**CpG2**	**CpG3**	**CpG4**	**CpG5**	**CpG6**
**HbA1c**	**0.0437**	**0.0075**	0.4165	0.7054	**0.026**	**0.0281**	0.448	0.1365	**0.0261**
**NAS**	**0.0479**	**0.0157**	0.3505	0.7686	**0.0089**	0.4483	0.5577	**0.0278**	0.2474
**Glucose**	0.0957	0.3029	0.5043	0.06	**0.0076**	0.7311	0.1573	0.3314	**0.0343**
**Insulin**	0.6487	0.3566	0.3958	0.7835	**0.0356**	0.1294	0.1905	**0.0338**	0.3006
**Creatinine**	0.7966	0.3052	0.3485	0.2044	0.8436	0.9028	0.913	0.5892	0.0869
**Cholesterol**	0.5533	0.9375	0.7201	0.2968	0.3105	0.1859	0.5029	0.224	0.2487
**Triglycerides**	0.1046	0.8258	0.4286	0.5428	0.3875	0.9572	0.5798	0.3977	0.3143
**HDL**	0.168	0.1968	0.5809	0.2768	0.6584	0.8278	0.9338	0.6668	0.8011
**LDL**	0.9417	0.8523	0.6762	0.1521	0.2851	0.24	0.187	0.5448	0.4918
**GGT**	0.2151	0.2149	0.506	0.5614	0.1237	**0.0033**	0.8175	0.0618	0.5998
**CRP**	0.9215	0.456	0.5914	0.0635	0.6828	0.361	0.4598	0.1028	0.1345
**TSH**	0.8431	0.2152	0.5616	0.6277	0.204	0.5466	0.6195	0.6308	0.7635
**AST**	0.5964	0.0779	0.7414	0.4694	0.1423	**0.0052**	0.7552	**0.006**	0.9224
**ALT**	0.2308	**0.0143**	0.2172	0.5307	0.2585	0.1391	0.644	**0.0019**	0.8689
**B12**	0.059	0.104	0.8028	0.0646	0.1914	0.1087	0.664	0.7995	0.9854
**Folic acid**	0.2554	0.2249	0.357	0.2952	0.9813	0.6466	0.1299	0.8208	0.2226
**let-7e-5p liver**	-	**0.0001**	**0.0257**	NA	NA	NA	NA	NA	NA
**IRS2 liver**	**0.0001**	-	NA	0.9061	0.071	0.6899	0.0597	**0.0025**	0.1514
**let7e-5p serum**	**0.0257**	NA	-	NA	NA	NA	NA	NA	NA
**cg25924746**	NA	0.9061	NA	-	0.523	**0.0259**	0.2263	0.3714	0.6534
**CpG2**	NA	0.071	NA	0.523	-	**0.0225**	**0.0000**	**0.0134**	**0.0399**
**CpG3**	NA	0.6899	NA	**0.0259**	**0.0225**	-	**0.0000**	**0.0412**	0.0883
**CpG4**	NA	0.0597	NA	0.2263	**0.0000**	**0.0000**	-	0.0838	**0.0389**
**CpG5**	NA	**0.0025**	NA	0.3714	**0.0134**	**0.0412**	0.0838	-	**0.0000**
**CpG6**	NA	0.1514	NA	0.6534	**0.0399**	0.0883	**0.0389**	**0.0000**	-

All significant p-values are indicated in bold. NA – not applicable.

ESM Table 4. q-values of all correlation analyses after correction for age, sex and BMI and corrected for multiple testing (q<0.15)

**P-values adjusted for age, sex and BMI and FDR-controlled; q < 0.15**									
	**let-7e-5p liver**	**IRS2 liver**	**let7e-5p serum**	**cg25924746**	**CpG2**	**CpG3**	**CpG4**	**CpG5**	**CpG6**
**HbA1c**	0.2123	**0.0578**	0.7734	0.8167	**0.0952**	**0.103**	0.8115	0.2502	0.1755
**NAS**	0.2123	**0.0722**	0.7734	0.8208	**0.0652**	0.6575	0.8115	**0.1021**	0.4559
**Glucose**	0.269	0.4387	0.7734	0.3555	**0.0652**	0.8465	0.4656	0.5207	0.1755
**Insulin**	0.8341	0.4825	0.7734	0.8208	**0.1097**	0.2782	0.4656	**0.1062**	0.494
**Creatinine**	0.9417	0.4387	0.7734	0.6529	0.8838	0.9458	0.9338	0.7201	0.2775
**Cholesterol**	0.8258	0.9375	0.7878	0.6529	0.4269	0.3407	0.8115	0.3791	0.4559
**Triglycerides**	0.269	0.9335	0.7734	0.772	0.5015	0.9572	0.8115	0.5468	0.494
**HDL**	0.3779	0.3695	0.7734	0.6529	0.7511	0.9106	0.9338	0.7335	0.9275
**LDL**	0.9417	0.9335	0.7878	0.6529	0.4181	0.4061	0.4656	0.7051	0.7213
**GGT**	0.4154	0.3695	0.7734	0.772	0.2721	**0.036**	0.8993	0.1511	0.8247
**CRP**	0.9417	0.5827	0.7734	0.3555	0.7511	0.5673	0.8115	0.2056	0.3699
**TSH**	0.9417	0.3695	0.7734	0.7986	0.3453	0.7516	0.8115	0.7304	0.9275
**AST**	0.8258	0.224	0.7878	0.772	0.2846	**0.038**	0.8745	**0.0328**	0.9663
**ALT**	0.4154	**0.0722**	0.7734	0.772	0.4063	0.2782	0.8115	**0.018**	0.9558
**B12**	0.2123	0.2658	0.8028	0.3555	0.3453	0.2656	0.8115	0.8208	0.9854
**Folic acid**	0.4179	0.3695	0.7734	0.6529	0.9813	0.8367	0.4656	0.8208	0.4559
**let-7e-5p liver**	-	**0.0025**	0.437	NA	NA	NA	NA	NA	NA
**IRS2 liver**	**0.0019**	-	NA	0.9061	0.1736	0.8432	0.3282	**0.018**	0.3702
**let7e-5p serum**	0.2123	NA	-	NA	NA	NA	NA	NA	NA
**cg25924746**	NA	0.9375	NA	-	0.6392	**0.1141**	0.4978	0.5447	0.8456
**CpG2**	NA	0.224	NA	0.772	-	**0.1141**	**0.0000**	**0.0591**	0.1755
**CpG3**	NA	0.8352	NA	0.3555	**0.0952**	-	**0.0000**	**0.1133**	0.2775
**CpG4**	NA	0.224	NA	0.6529	**0.0000**	**0.0000**	-	0.1844	0.1755
**CpG5**	NA	**0.0283**	NA	0.7427	**0.0739**	0.1511	0.3688	-	**0.0000**

**CpG6**	NA	0.3483	NA	0.7986	**0.1097**	0.2428	0.2849	**0.0000**	-

All significant q-values (q<0.15) are indicated in bold. NA – not applicable.

ESM Table 5: p-values and q-values (FDR 5%) for the correlation analysis between hepatic IRS2 expression and downstream genes in the insulin signaling pathway.

**Correlation with hepatic IRS2 expression**		
	p-value	q-value
Let-7e-5p	**0.00016918**	**0.000676723**
ACACB	**0.04657059**	0.069855889
SCD	**0.00000354**	**0.0000212671**
ACACA	**0.000000789**	**0.00000947028**
ELOVL6	0.08399091	0.111987881
FASN	**0.00474068**	**0.014222032**
PNPLA2	**0.02053141**	**0.035196711**
VAT_IRS2	0.34826574	0.417918884
FOXO1	0.66504445	0.665044452
G6PC	0.55201915	0.602202708
PCK1	**0.01119849**	**0.026876371**
PDK1	**0.01714382**	**0.034287644**

All significant p-values ($p<0.05$) and q-values ($q<0.05$) are indicated in bold.

ESM Figures

ESM Fig. 1

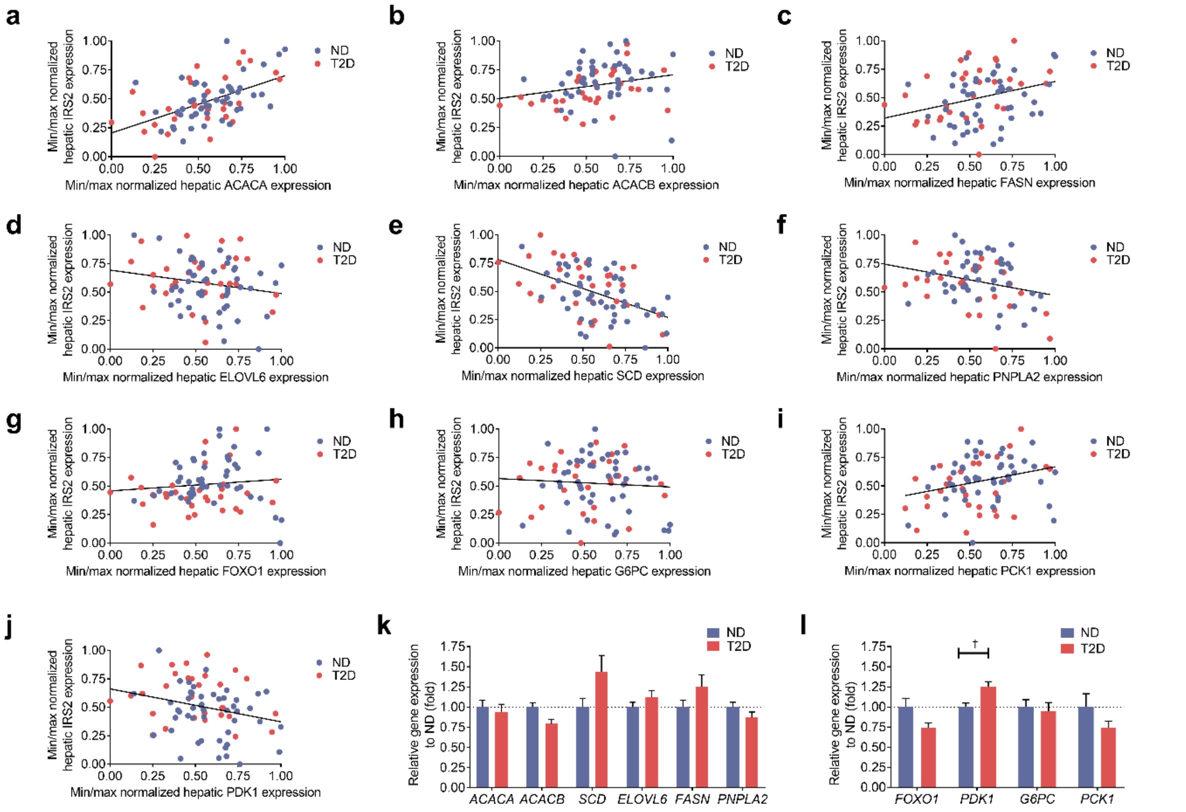

ESM Figure 1: Associations between altered *IRS2* expression and the expression of genes involved in lipogenesis (*ACACA, ACACB, FASN, SCD*), lipolysis (*PNPLA2*), glucose metabolism (*FOXO1, G6PC, PCK*) and insulin signal transduction (*PDK1*).

A-j) Correlation between hepatic IRS2 expression and downstream genes of insulin signalling. A) ACACA (p=7.89E-07, q=9.47E-06, r=0.52, n=81), c) FASN (p=0.0047, q=0.014, r=0.31, n=81) and i) PCK1 (p=0.0112, q=0.0267, r=0.28, n=80) correlate positively with *IRS2* expression, whereby e) SCD (p=3.54E-06, q=2.127E-05, r=-0.49, n=81), f) PNPLA2 (p=0.0205, q=0.0352, r=-0.27, n=72) and j) PDK1 (p=0.0171, q=0.0343, r=-0.27, n=78) correlate negatively with *IRS2* expression. Expression of b) ACACB (p=0.0466, q=0.07, r=0.23, n=81) g) *FOXO1* (p=0.6650, q=0.665, n=79) and h) *G6PC* (p=0.5520, q=0.6022, n=80) does not correlate with *IRS2* expression. These results indicate that changes in *IRS2* expression are associated to changes of downstream genes of lipid and glucose metabolism. A comparison between type 2 diabetic and non-diabetic subjects regarding genes of lipid (k) and glucose metabolism (l) reveals that only *PDK1* is significantly 1.25-fold upregulated in type 2 diabetes (l, p=0.0046, †p_a=0.0131). For Pearson correlation analysis, mRNA expression was normalized to the expression of a housekeeper gene (dC_t). For visualization, these dC_t values were normalized by min/max normalization $(1 - (x_i - x_{min})/(x_{max} - x_{min}))$ to a scale from 0 (minimum expression) to 1 (maximum expression) and indicated as normalized expression on the axis. Differences in gene expression for both subgroups were normalized to the ND group by the $2^{-\Delta\Delta Ct}$ method and

indicated as relative difference (fold). Statistics were performed by two-sided Student's t-test for unadjusted p values and by logistic regression for age, sex and BMI adjustment (p_a). X_i, current ΔCt value of a specific gene; x_{min}, minimum ΔCt value of all measurements of a specific gene; x_{max}, maximum ΔCt value of all measurements of a specific gene. Error bars indicate mean +/- SEM, †p_a<0.05 and q<0.05 compared to ND.

ESM Fig. 2

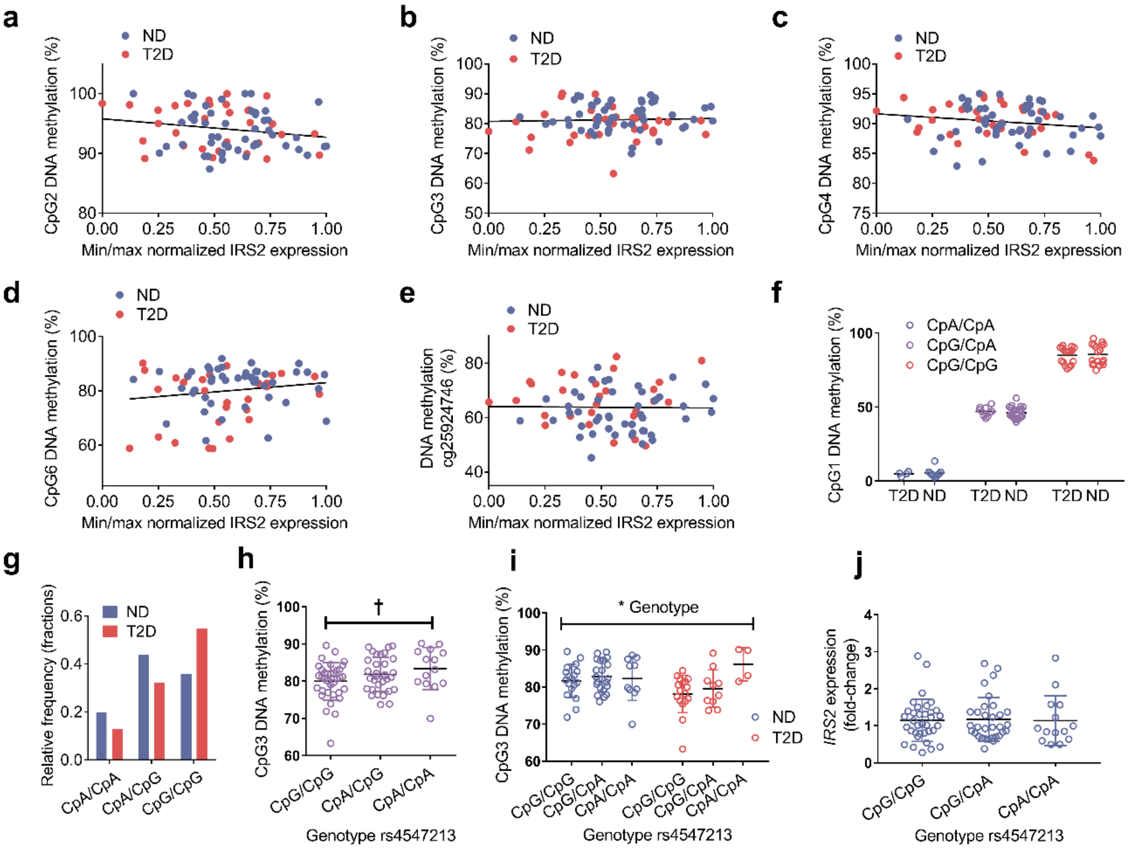

ESM Fig. 2: Correlation plots between DNA methylation and hepatic *IRS2* expression, as well the influence of the polymorphism rs4547213 on DNA methylation at CpG1, DNA methylation CpG3 and on *IRS2* expression.

Correlation analysis between hepatic *IRS2* expression and hepatic DNA methylation at (a) CpG2 (p_a=0.0710, q= 0.224, r=-0.1849, n=81), (b) CpG3 (p_a=0.6899, q=0.8352, r=0.0406, n=81), (c) CpG4 (p_a=0.0600, q=0.224, r=-0.1734, n=81), (d) CpG6 (p_a=0.1514, q=0.3483, r=0.1638, n=76), and (e) at cg25924746 (p_a=0.9061, q=0.9375, r=-0.0141, n=75). Methylation at the CpG-SNP cg12195446-rs4547213, here referred as CpG1, is not altered between type 2 diabetic and non-diabetic subjects after stratification for the genotype at rs4547213 (f). The distribution of the polymorphism suggests the G allele to represent the risk allele but a chi-squared test (α < 0.05) and logistic regression revealed no significant association to the incidence of T2D in this cohort (g). The minor allele A of rs4547213 has a frequency (MAF) of about 37 % in the whole cohort (n=81), which is comparable to known frequencies from British or Finish cohorts (GBR MAF 36.8 % and FIN MAF 31.3 % from 1000 Genomes Project). (h) The polymorphism rs4547213 is significantly associated to changes in DNA methylation at CpG3

within the SP1 recognition motif ($p=0.0235$, †$p_a=0.0348$) The effect of the polymorphism on DNA methylation was assessed by logistic regression either without addition of age, sex and BMI (p) or with addition of these cofactors (p_a). (i) The CpG genotype at rs4547213 is associated to a lower CpG3 methylation in type 2 diabetic (T2D) subjects (*$p=0.0350$, two-way ANOVA, a post-hoc test by comparing each cell means regardless of rows and columns and adjustment of p-values by the FDR method of Benjamini and Hochberg). The genotype itself is not associated to the incidence of T2D ($p=0.1249$) Group comparison shows a significant difference between homozygous CpA and CpG genotype in individuals with type 2 diabetes ($p=0.0035$, $q=0.0262$). The difference between homozygous CpA and heterozygous CpA/CpG genotype ($p=0.0255$, $q=0.1017$) becomes insignificant after applying a FDR threshold of $q<0.05$. (j) An expression quantitative trait loci (eQTL) analysis including age, sex and BMI as co-factors reveals no association between the genotype at rs4547213 and altered *IRS2* gene expression ($p>0.05$). This is indicated by no difference in fold change expression relative to non-diabetic major allele carriers (CpG/CpG) after stratification for the genotype. Correlation analysis was performed on DNA methylation and dC_t values first by Pearson correlation (p) and additionally by linear regression models to adjust for age, BMI and sex (p_a). For visualization, gene expression was normalized by min/max normalization $(1 – (x_i – x_{min})/(x_{max} - x_{min}))$ to a scale from 0 (minimum expression) to 1 (maximum expression) and indicated as normalized expression on the axis. To assess the influence of the polymorphism and disease state, a two-way ANOVA was performed, *$p<0.05$. The influence of the polymorphism on disease incidence was calculated by logistic regression using age, BMI and sex as co-factors. *IRS2* expression was calculated by the $2^{-\Delta\Delta Ct}$ method and indicated as fold. The mean +/- SD for methylation and the mean fold-change +/- SEM for gene expression is additionally shown. X_i, current ΔCt value of a specific gene; x_{min}, minimum ΔCt value of all measurements of a specific gene; x_{max}, maximum ΔCt value of all measurements of a specific gene.

ESM Fig. 3

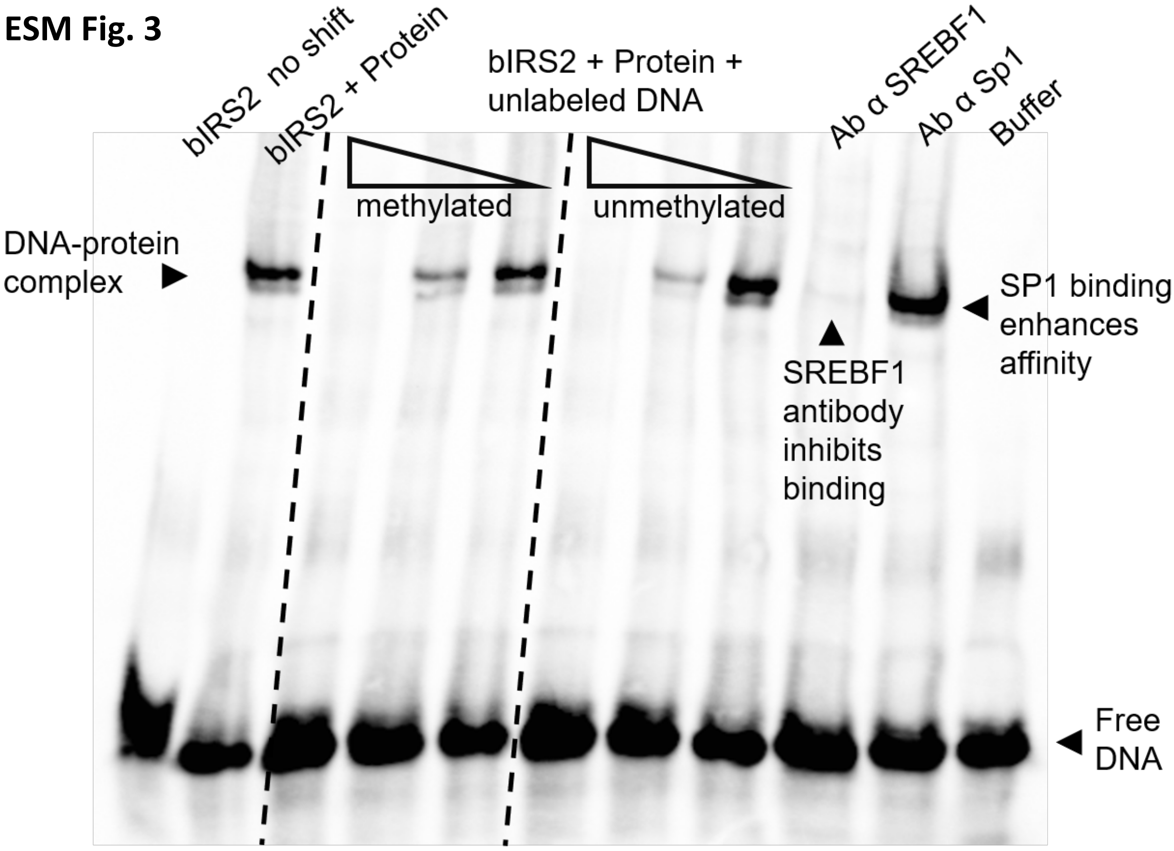

ESM Fig. 3: Complete blot of the electrophoretic mobility shift assay.

Electrophoretic mobility shift assay shows a shift of biotinylated DNA oligonucleotides containing CpG1 to CpG6 after incubation with nuclear Extract of HepG2 cells (bIRS2+Protein, lane 2) which can be erased after incubation with different concentrations (12.5 pmol, 1.25 pmol, and 0.125 pmol, indicated as gradient) of unlabelled DNA (lanes 3-5 for unlabelled fully-methylated DNA and lanes 6-8 for unlabelled un-methylated DNA). Incubation of the binding reaction with antibody against SREBF1 (Ab α SREBF1, lane 9) or Sp1 (Ab α SP1, lane 10) for confirmation of proposed transcription factors results into intensity change of shifted biotinylated DNA. Control for nuclear extraction buffer only (Buffer, lane 11) shows no background signals. It is not possible to quantify the effects of competitive binding for fully-methylated and un-methylated DNA accurately due to signal saturation of the free DNA which serves as background control. The dashed lines indicate the cropped part which is missing in Fig. 3d.

ESM References

1. Vilahur N, Baccarelli AA, Bustamante M, et al (2013) Storage conditions and stability of global DNA methylation in placental tissue. Epigenomics 5(3):341–348. https://doi.org/10.2217/epi.13.29

2. Li Y, Pan X, Roberts ML, et al (2018) Stability of global methylation profiles of whole blood and extracted DNA under different storage durations and conditions. Epigenomics 10(6):797–811. https://doi.org/10.2217/epi-2018-0025

3. Andersen CL, Jensen JL, Ørntoft TF (2004) Normalization of real-time quantitative reverse transcription-PCR data: a model-based variance estimation approach to identify genes suited for normalization, applied to bladder and colon cancer data sets. Cancer Res 64(15):5245–5250. https://doi.org/10.1158/0008-5472.CAN-04-0496

4. Klug M, Rehli M (2006) Functional analysis of promoter CpG methylation using a CpG-free luciferase reporter vector. Epigenetics 1(3):127–130. https://doi.org/10.4161/epi.1.3.3327
